# Effect of Auditory Maturation on the Encoding of a Speech Syllable in the First Days of Life

**DOI:** 10.3390/brainsci11070844

**Published:** 2021-06-25

**Authors:** Laís Ferreira, Piotr Henryk Skarzynski, Magdalena Beata Skarzynska, Milaine Dominici Sanfins, Eliara Pinto Vieira Biaggio

**Affiliations:** 1 Speech Therapy Department, Federal University of Santa Maria, Santa Maria 97105-900, Brazil; eliarapv@yahoo.com.br; 2 Postgraduate Program in Human Communication Disorders, Federal University of Santa Maria, Santa Maria 97105-900, Brazil; 3 Institute of Physiology and Pathology of Hearing, 02-042 Warsaw, Poland; p.skarzynski@inz.waw.pl; 4 Department of Heart Failure and Cardiac Rehabilitation, Medical University of Warsaw, 02-091 Warsaw, Poland; 5 Institute of Sensory Organs, 05-830 Warsaw, Poland; m.skarzynska@csim.pl; 6 World Hearing Center, Institute of Physiology and Pathology of Hearing, 02-042 Kajetany, Poland; 7 Center of Hearing and Speech Medincus, 05-830 Kajetany, Poland; 8 Faculty of Medical Science, State University of Campinas, Campinas 13083-887, Brazil; msanfins@uol.com.br; 9 Advanced Electrophysiology and Neuroaudiology Center, Department of Electrophysiology, São Paulo 04515-030, Brazil

**Keywords:** hearing, evoked potentials, auditory, child development, infant

## Abstract

(1) Background: In neonates and infants, the physiological modifications associated with language development are reflected in their Frequency Following Responses (FFRs) in the first few months of life. (2) Objective: This study aimed to test the FFRs of infants in the first 45 days of life in order to evaluate how auditory maturation affects the encoding of a speech syllable. (3) Method: In total, 80 healthy, normal-hearing infants, aged 3 to 45 days old, participated in this study. The sample was divided into three groups: GI, 38 neonates from 3 to 15 days; GII, 25 infants from 16 to 30 days; and GIII, 17 infants from 31 to 45 days. All participants underwent FFR testing. Results: With age, there was a decrease in the latency of all FFR waves, with statistically significant differences among the groups studied for waves V, A, E, F, and O. The mean amplitudes showed an increase, with a statistically significant difference only for wave V. The slope measure increased over the 45 days, with a statistically significant difference between GIII and GI and between GIII and GII. (4) Conclusions: The encoding of a speech sound changes with auditory maturation over the first 45 days of an infant’s life.

## 1. Introduction

In infants, the adequate assessment of hearing is indispensable, since auditory function is one of the prerequisites for the development of an individual’s social communication skills.

To achieve adequate language development, an infant needs, in addition to normal peripheral hearing, coordinated function of the entire central auditory pathway [1]. While perception of sound begins in the brainstem, more complex auditory skills depend on the subcortex and cortex [2].

The perception of speech involves representing complex sounds linguistically and identifying general patterns of auditory features. Speech and hearing both involve the complex processing of acoustic information, and both depend on each other [3,4]. Linguistic experience, through contact with the native language from birth, promotes an infant’s speech perception, language, and cognition [5].

The Frequency Following Response (FFR) is an auditory evoked potential, the recording of which makes it possible to investigate the way in which the neural encoding of speech develops [2,6,7], thereby allowing early language and developmental skills to be monitored [2,7,8].

In neonates and infants, the physiological modifications associated with language development are reflected in the FFR responses over the first few months of life [9]. These responses allow infants with delays in speech perception, or who are at risk of developing such language anomalies, to be identified, and such screening provides a basis for early intervention [10,11,12]. In this way, the FFR appears to be a promising tool as a predictor of future speech and language problems [7,13].

Human communication is based on a direct connection with auditory experience, and the underlying processes involve high levels of neural activity [14]. Development of the auditory nervous system is shaped by auditory experiences at the beginning of life, with the central structures undergoing prolonged development in response to diverse stimuli [1,2]. Understanding the physiological alterations that take place during maturation of the auditory system in the first days of life, and how they link with language skills, is important for identifying those infants at risk who may require early intervention.

In this scenario, the FFR represents a strategy to assess the encoding of speech sounds during early life [2,7]. This research aims to assess auditory maturation in terms of changes in the encoding of a speech syllable during the first 45 days of life and to derive normative values of the FFR for this population.

## 2. Materials and Methods

### 2.1. Ethics

This research had an observational, analytical, descriptive, and quantitative cross-sectional design. It was approved by the Research Ethics Committee of our institution (number CAAE 81117517.0.0000.5346 and opinion 2.538.043). Those responsible for the infants signed an informed consent form. The norms and regulatory guidelines for research with human beings of Resolution 466/2012 of the Brazilian National Health Council were respected.

### 2.2. Participants

There were 80 healthy, normal-hearing infants, of both genders (45 males and 35 females), aged between 3 and 45 days of life, who participated in the study. None of them had any Risk Indicator for Hearing Impairment (RIHI) [15], middle ear problems, or evident neurological conditions. All were recruited from the Neonatal Hearing Screening (NHL) program of a public university hospital.

All infants included in the sample were born at term (39 to 40 weeks); had a normal NHL score using the evoked otoacoustic emission procedure [15]; had an Apgar score >8 after 1 and 5 min from birth; and normal (for their age) absolute latencies of waves I, III, and V and interpeak intervals I–III, III–V, and I–V in an Auditory Brainstem Response (ABR) test obtained with a click of 80 dBnHL [16]. The ABR was performed in order to guarantee the integrity of the auditory pathway at the brainstem level.

In order to assess the effect of auditory maturation in terms of FFR responses, the sample was divided into three groups according to age: Group I (GI), 38 neonates from 3 to 15 days of age; Group II (GII), 25 infants from 16 to 30 days of age; and Group III (GIII), 17 infants from 31 to 45 days. The groups had mean ages of 9.5, 23, and 37 days, respectively.

### 2.3. Research Procedure

The FFR assessment was performed using the Smart EP module from Intelligent Hearing Systems (Miami, FL, USA). Disposable surface electrodes were used, and the infant’s skin was sanitized with abrasive gel (Nuprep). The electrodes were positioned at Fz, Fpz, M1, and M2. Impedance was maintained below 3k ohms, with the difference between the electrodes kept below 2k ohms.

Parameters established in the literature were followed [8,17]. The syllable [da], 40 ms in duration, was presented monaurally to the right ear at an intensity of 80 dBnHL. The presentation rate was 10.9/s with an analysis window of 80–100 ms; the polarity alternated, and a high-pass filter of 100 Hz and a low-pass filter of 2000 Hz were used.

The infants underwent two scans of 3000 sweeps. The recordings of both stimulations were summed to generate a resultant trace, which was used in data analysis. The following parameters were extracted from the recordings: absolute latency and amplitude of waves V, A, C, D, E, F, and O and the V–A slope measure (measured by the software itself). The marking of the waves was checked by 3 examiners with experience in auditory electrophysiology.

When capturing FFR responses, the infants remained asleep, comfortably accommodated in the lap of their carer. Data collection took an average of 30 min. Tests that contained more than 5% artifacts were excluded.

### 2.4. Data Analysis

The values extracted from the examinations were assembled in a spreadsheet. To determine whether the sample distribution of each group was normal or not, a Shapiro–Wilk test was used. If the analyzed variables did not have a normal distribution, statistical analysis between the groups was performed using a Kruskal–Wallis test, a nonparametric statistical test equivalent to a unidirectional ANOVA. The confidence level was set at 95%.

## 3. Results

**Figure 1:**
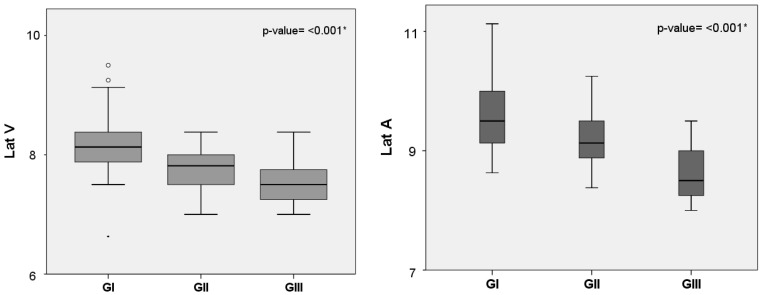

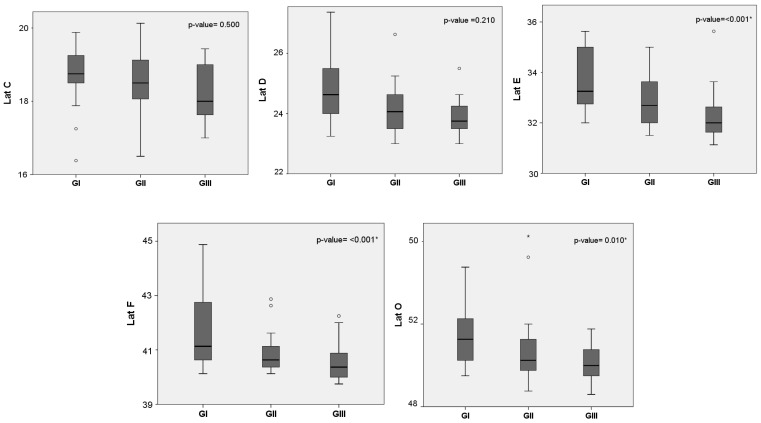
Absolute latency (ms) of the different Frequency Following Response waves in three groups of infants in the first days of life. GI (*n* = 38) mean age: 9.5 days; GII (*n* = 25) mean age: 23 days; and GIII (*n* = 17) mean age: 37 days. Statistical test: Kruskal–Wallis. Lat = latency; * indicates a statistically significant difference.

The results showed FFR waves present in the first days of life. The mean latencies for the three groups (GI, GII, and GIII) were identified and analyzed. Data indicated that auditory maturation takes place during the first 45 days of the infant’s life. There was a numerical decrease in the absolute latency of all FFR waves as the infants became older, with statistically significant differences for waves V, A, E, F, and O (Figure 1).

In the paired comparison analysis, a statistically significant difference was observed between the three groups for wave A (*p* = 0.016 *; *p* ≤ 0.001 *; *p* = 0.036 *). For wave O, a difference (*p* = 0.001 *) was found between GI and GII. For waves V (*p* ≤ 0.001*), E (*p* < 0.001 *), and F (*p* = 0.001 *), statistically significant differences were obtained between GI and GIII.

In terms of mean amplitudes, it was found that the values of all FFR waves increased between GI and GIII. However, as shown in Table 1, this difference was statistically significant only for wave V (*p* = 0.001 *) between GI and GIII.

**Figure 2:**
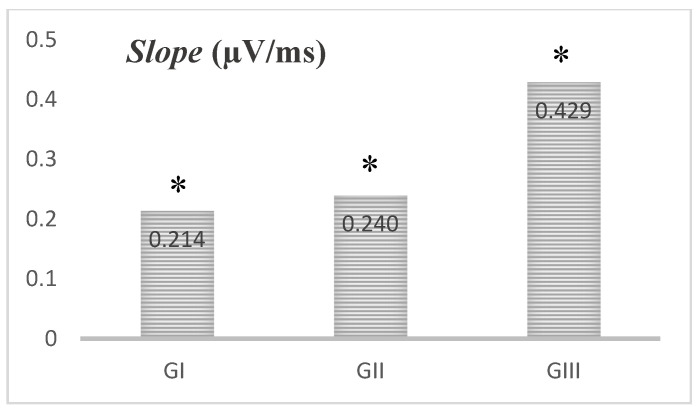
Slope of the VA complex extracted from the Frequency Following Response assessment in the three groups surveyed (*n* = 80). Statistical test: Kruskal–Wallis; * indicates a statistically significant difference between GI and GIII (*p* ≤ 0.001 *) and between GI and GII (*p* ≤ 0.001 *).

Another measure analyzed was the slope. The slope is the measure, in µV/ms, between peak V and peak A, which increased during the 45 days of life, as shown in Figure 2. There was a statistically significant difference between GI and GIII (*p* ≤ 0.001 *) and between GI and GII (*p* ≤ 0.001 *).

## 4. Discussion

This study presents data on the encoding of a speech syllable in the first 45 days of life. The data showed that the physiological changes involved in the maturation process of the auditory pathway are reflected in the FFR responses. Changes were observed in terms of a decrease in neural conduction time, and an increase in the amplitude of the response and the VA slope, the latter reflecting an improvement in the neural synchronicity of the response generators.

The spectral encoding of sound is a major part of speech perception [9]. Further, the perception of speech is a fundamental skill in the development of language in children [3]. Researchers have observed, through brain activity evoked by speech sounds, that areas associated with language are activated in infants well before the onset of speech production. Functional MRI data demonstrate that infants possess the ability to perceive speech sounds [18]. Linguistic experience, acquired from contact with the native language from birth, promotes speech perception, language, and cognition in infants [5].

Since testing found FFR responses in the first 45 days of life, this research supports the idea that the encoding of speech begins in this period. Other research performed on neonates has also observed FFR responses in the initial days of life [11,19,20,21].

A possible explanation for the presence of FFR waves in the first few days of life is prenatal auditory experience. This hypothesis is consistent with research that has shown that prenatal experience has effects on the development of the central auditory system [2,9] and on auditory perception [22].

Authors who have studied the effects of prenatal linguistic experiences on the neural processing of speech have verified that the brain of the neonate, from 0 to 3 days of life, has distinctly different electrophysiological patterns in response to familiar speech and to unknown speech, indicating that the human brain receives and processes stimulation during the gestational period [23]. Therefore, auditory stimulation during the prenatal period already has an impact on the early speech processing of the neonate.

Several studies carried out with the FFR have seen a decrease in latency as age increases [1,9,10]. In infants, signs of auditory maturation were observed in FFR responses when comparing the recordings of infants aged 1 and 3 months [10], and those aged 3 to 5, 6 to 10, and 3 to 10 months [9]. The present study provides further evidence regarding auditory maturation in the first 45 days of life (Figure 1). In agreement with the studies cited above, this research also found a decrease in the neural conduction time with age. Thus, already in the first days of life, one can infer that the neural activity of an infant’s auditory pathway is intense and stimulus dependent.

The pattern observed for the amplitude values was similar to that obtained for latency. There was an increase in the mean amplitudes during the first 45 days of life, especially in wave V (Table 1). The amplitude values represent the magnitude and robustness of the neural response. The amplitude of the V–A complex represents a response to the onset burst, the consonant plosive /d/ [24]. In another study with neonates, the values found were generally similar to those of this study, particularly for waves A, C, and E [20]. Another finding that demonstrated the effect of maturation on response amplitudes was from a study on children aged 3–4 years old, which verified that the highest amplitudes came from waves V, A, and F and increased with age [8].

It is believed that both the decrease in neural conduction time and the increase in the magnitude of the responses observed over the course of 45 days are influenced by myelination and modification in synaptic function [9]. Both result from the functional maturation of the auditory pathway and the subject’s auditory experiences, as previously reported.

The early stages of brain maturation affect cerebral potentials and vulnerability, and are, therefore, considered important for development [25]. In this context, it is known that the cortex and subcortical gray matter nuclei develop in utero. At birth, the brain of a newborn is one-fourth to one-third of the volume of an adult, so the infant brain must develop enormously and specialize according to genetics and the environment [25].

The central auditory nervous system undergoes prolonged development, part of which is maturation of the auditory pathway. The neural origins of the FFR waves are not yet clear, but one study indicates that the responses are influenced by the cochlear nucleus, inferior colliculus, medial geniculate body, and auditory cortex [26]. Another investigation which studied the myelination process of these structures verified that this process occurs gradually, with the authors observing that the cochlear nucleus, the superior olivary complex, and the lateral lemniscus undergo an increase in myelination up to the 13th week of life, the inferior colliculus up to the 39th week [27], the medial geniculate body up to the 10th week, and the splenius of the corpus callosum up to the 16th postnatal week [27].

The experiences in the infantile period are of great importance for the development and formation of the brain, since the human brain continues to modify and adapt during this period [28]. Subcortical auditory activity is molded depending on the experiences to which the child is exposed throughout life [1]. Linguistic experiences raise neural activity and increase the number of connections to developing central structures [14,25]. Thus, such experiences, among them auditory ones, combine and shape the individual’s auditory pathways, directly affecting the auditory nervous system’s development [1] and the myelination process.

Myelin makes an important contribution to increasing the speed of nerve conduction, and is essential for auditory function [14]. In this way, linguistic experiences result in modifications to neural networks specialized for encoding speech [29]. Thus, as age advances, there is greater exposure to sounds and higher neural activity. The observed decrease in FFR wave latency with age makes it possible to understand how the auditory experiences received in the first few days of life have already brought about physiological modifications observable in the FFR.

The slope parameter is considered a measure of the temporal synchronicity of neural response generators, and reflects a relation between the time and magnitude of a neural response [30]. As it depends on both latency and amplitude, the slope parameter also showed a reduction in its mean value over the 45 days (Figure 2). In considerably older infants, from 3 to 10 months, the slope measure is also seen to improve with age [9]. These results enable us to infer that auditory experiences associated with the auditory pathway maturation result in an improvement in the synchronicity of the generator of the V–A complex.

The findings of this research, alongside those of other authors [1,9,27,31], indicate that an increase in myelination associated with the maturation of the auditory nervous system, together with auditory experiences, results in a reduction in neural conduction time and an improvement in the processing of acoustic information. The present study indicates FFR assessment as a potential tool that may help measure the maturation processes mentioned.

Given the effect of the maturation process on the auditory pathway throughout the lifespan [1], it is appreciated that making an assessment in a particular case requires the use of good standard values for the age being considered. For FFR assessment, two pieces of research reported reference normative data for the neonatal population [20,21]. The values observed in this research for latency, amplitude, and slope are similar to those found in the literature when using Navigator Pro-Biologic equipment [20]. One piece of research also used the Smart EP module from Intelligent Hearing Systems for the FFR assessment of neonates [21]. However, this study is pioneering in using the Smart EP module to provide reference values for latency, amplitude, and slope parameters for infants.

If the FFR is to be used in clinical practice as a potential marker of future language impairment, it is necessary to establish reference values for each age range. This study is the first to provide reference values for latency, amplitude, and slope for infants using the Smart EP module. However, it will only be possible to identify normal and altered test results if there are standards with a strong scientific basis.

This research confirmed that FFR responses change systematically with age, indicating functional maturation of the central auditory system [1,9,10]. However, it is notable that this effect is seen in just the first few days of an infant’s life. Taken together, these results support the idea that the assessment of this auditory evoked potential provides insight into the neurophysiological modifications arising during the early developmental process.

## 5. Conclusions

The data from this research show that there is an effect of auditory maturation on the encoding of speech sounds in the first 45 days of an infant’s life, indicating that neurophysiological modifications at the subcortical and cortical level occur rapidly. Changes were observed in terms of a decrease in neural conduction time, and an increase in the amplitude of the response and the VA slope, the latter reflecting an improvement in the neural synchronicity of the response generators.

This study also allows reference normative values for FFR assessments in infant populations to be set.

## 6. Patents

This section is not mandatory but may be added if there are patents resulting from the work reported in this manuscript.

## Figures and Tables

**Table 1:** Amplitude (μV) of the Frequency Following Response waves in the first days of life (*n* = 80).

	Age (Days)	V	A	C	D	E	F	O
Mean	3 to 15	0.14	−0.16	−0.09	−0.12	−0.19	−0.14	−0.16
amplitude	16 to 30	0.16	−0.15	−0.08	−0.10	−0.16	−0.13	−0.15
(µV)	31 to 45	0.22	−0.23	−0.09	−0.13	−0.19	−0.19	−0.20
*p*-value		0.001 *	0.071	0.085	0.363	0.276	0.092	0.060

Statistical test: Kruskal–Wallis; * indicates a statistically significant difference (between three and 15 days, and 31 and 45 days).
